# Identification of drug responsive enhancers by predicting chromatin accessibility change from perturbed gene expression profiles

**DOI:** 10.1038/s41540-024-00388-8

**Published:** 2024-05-30

**Authors:** Yongcui Wang, Yong Wang

**Affiliations:** 1 State Key Laboratory of Phytochemistry and Plant Resources in West China, Kunming Institute of Botany, Chinese Academy of Sciences, Kunming, 650201 China; 2 CEMS, NCMIS, HCMS, MDIS, Academy of Mathematics and Systems Science, Chinese Academy of Sciences, 100190 Beijing, China; 3 Key Laboratory of Systems Biology, Hangzhou Institute for Advanced Study, University of Chinese Academy of Sciences, Chinese Academy of Sciences, Hangzhou, 330106 China

**Keywords:** Computational biology and bioinformatics, Cancer

## Abstract

Individual may response to drug treatment differently due to their genetic variants located in enhancers. These variants can alter transcription factor’s (TF) binding strength, affect enhancer’s chromatin activity or interaction, and eventually change expression level of downstream gene. Here, we propose a computational framework, PERD, to Predict the Enhancers Responsive to Drug. A machine learning model was trained to predict the genome-wide chromatin accessibility from transcriptome data using the paired expression and chromatin accessibility data collected from ENCODE and ROADMAP. Then the model was applied to the perturbed gene expression data from Connectivity Map (CMAP) and Cancer Drug-induced gene expression Signature DataBase (CDS-DB) and identify drug responsive enhancers with significantly altered chromatin accessibility. Furthermore, the drug responsive enhancers were related to the pharmacogenomics genome-wide association studies (PGx GWAS). Stepping on the traditional drug-associated gene signatures, PERD holds the promise to enhance the causality of drug perturbation by providing candidate regulatory element of those drug associated genes.

## Introduction

The completion of the Human Genome Project enables a good understanding of coding regions. This allows pharmacogenomics studies to have focused on the drug-associated gene signatures, and many computational models predicted potential drug-gene associations and built user-friendly web server on a large scale^1–3^. However, the coding region of the human genome only accounts for about 2% of the entire genome^4–6^ and the remaining region (non-coding region) has been demonstrated to play important roles in regulating transcriptional and non-transcriptional processes^7–10^. Genome-wide association studies (GWAS) have revealed numerous diseases and phenotypic traits associated single nucleotide variants (SNVs) located in non-coding regions^11–14^. In addition to disease-related genetic variants, GWAS has uncovered numerous variants associated with drug sensitivity, with a majority of them situated within non-coding regulatory elements^15,16^. Those variants may play a very important role in explaining individual’s heterogenous response to drug treatment in personalized medicine^17,18^.

In addition, several successful biological experiments have suggested the influence of nucleotide changes located in gene regulatory elements on drug sensitivity^19^. For instance, a single nucleotide polymorphism (SNP) in the promoter of gene vitamin K epoxide reductase complex subunit 1 (*VKORC1*), fundamentally influenced the individual’s reaction to the anticoagulant warfarin^20^; a SNP located in the enhancer of several solute carrier family (*SLC*) drug transporters was reported to link to the clearance of methotrexate (*MTX*)^21^. In addition, variants within the regulatory regions of drug metabolizing enzymes and drug transport proteins will also affect the therapeutic effect of drugs. For example, SNPs in the promoter region of the drug-metabolizing enzyme *CYP3A4* will cause changes in the expression level of the gene, which in turn will change the efficacy of the drug^22^. Computational model also suggested the close relationships between non-coding regions and small molecules^23^. Therefore, systematic identification of regulatory elements related to drug sensitivity is of great significance for enhancing the causal understanding of drug-associated gene and revealing genetic variations that could intervene the patient’ response to drug treatment.

In order to identify drug responsive regulatory elements, one way is to assess the alterations in chromatin activity resulting from drug perturbations. We noticed that high-throughput sequencing technologies, such as Chromatin Immunoprecipitation Sequencing (ChIP-seq)^24^, not only effectively determine the position of a large number of regulatory elements in the genome but also improve the annotation of the function of these elements^25^. High-throughput chromosome conformation capture (Hi-C)^26^, split-pool recognition of interactions by tag extension (SPRITE)^27^, genome architecture mapping (GAM)^28^, chromatin interaction analysis by paired-end tag sequencing (ChiA-PET)^29^, could detect chromatin interactions in the mammalian nucleus, and the pulldown methods such as HiChIP^30^, PLAC-seq^31^, etc., integrated ChIP-seq and Hi-C, to reveal the interactions between regulatory elements and their targets. Encyclopedia of DNA elements (ENCODE) database has curated and deposited all these high-throughput sequencing data to identify functional elements within the human genome^2^. It includes ChIP-seq data across over 2700 cell lines, DNase-seq data across over 500 cell lines, and RNA-seq for over 200 cell lines. However, perturbed chromatin activity profiles, where chromatin activity is measured after a drug perturbation, are seriously inadequate compared to several main large scale perturbed gene expression profile datasets. This greatly hinders the progress to directly connect the causal perturbations to their regulatory element activity consequences.

In this paper, we aim to overcome the lack of perturbation chromatin activity data by computationally predicting chromatin accessibility via paired expression and chromatin accessibility data accumulated in ENCODE and ROADMAP. These valuable paired data offer machine learning a great gold standard dataset to predict regulatory elements’ activity from gene expression. For instance, Zhou et al., have developed a computational framework (BIRD) to predict the genome locus chromatin accessibility measured by DNase I hypersensitivity (DH) from biological sample’s transcriptome^32^. The application of BIRD in predicting TF-binding sites (TFBSs), turning publicly available gene expression samples in Gene Expression Omnibus (GEO) into a regulome database, which contains the regulatory element activities^32^. Inspired by this application, the drug-dependent enhancers could be detected by utilizing main large scale perturbation gene expression profile datasets, predicting regulatory element’s activity upon drug treatment, and finding enhancers that display the significantly different chromatin accessibility after drug treatment.

In particular, a computational framework, called PERD, was developed to **P**redict the **E**nhancers **R**esponsive to **D**rug by assessing their chromatin accessibility changes after drug treatment. A regularized regression model with potential TF-enhancer and enhancer-gene interactions as constraints was constructed to predict enhancer’s chromatin accessibility. The validation on paired DNase-seq and RNA-seq data from ENCODE and Roadmap indicated the feasibility of using transcriptome of enhancer’s associated genes and binding TFs to predict its chromatin accessibility. Then, the enhancer chromatin accessibility before/after given drug treatment was predicted from perturbed gene expression profile datasets^33,34^ and compared. The enhancers that display the significantly different chromatin accessibility were output as the drug responsive enhancers. As a pilot study, the drug responsive enhancers were then related with TF motifs and pharmacogenetics (PGx), to identify the variants related to drug perturbations.

## Results

**Fig. 1:**
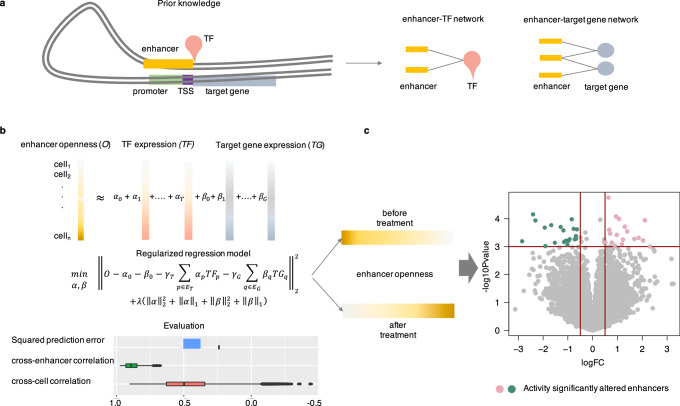
The framework of PERD. **a** Construction of the TF-enhancer and enhancer-target gene network from ChIP-seq experiments and curated database as prior knowledge. **b** Construction of a regularized regression model to predict enhancer’s chromatin activity from the expression of its binding TFs and associated target genes. The prediction model was evaluated by squared prediction error, cross-cell, and cross-enhancer P-T correlations. **c** Prediction of the drug responsive enhancers by comparing the enhancer’s chromatin activity before/after drug treatment. The enhancers with significantly different chromatin activities (large fold change and small *p* value) were output as the drug responsive enhancers.

PERD was proposed to identify drug responsive enhancers by predicting the changes in enhancers’ chromatin accessibility from perturbed gene expression profiles. In particular, the priority knowledge about enhancer associated genes and TF binding regions were firstly collected to construct the enhancer-gene and TF-enhancer network, respectively (Fig. 1a). Then, a regularized regression model was established to predict enhancer’s chromatin accessibility from the transcriptional expression of its associated genes and binding TFs (Fig. 1b). The model was trained on the paired DNase-seq and RNA-seq data in ENCODE. The drug responsive enhancers were revealed through identifying enhancers that display significantly changed chromatin accessibilities after drug treatment (Fig. 1c). More detail of PERD will be explained in Methods.

Several validations were conducted to evaluate the PERD model. It was found that PERD can efficiently reveal enhancer’s chromatin accessibility on the basis of transcriptome data and identify non-coding regions closely related with drug perturbation.

### Predicting enhancer’s chromatin accessibility from the expression level of its associated genes and binding TFs

**Fig. 2:**
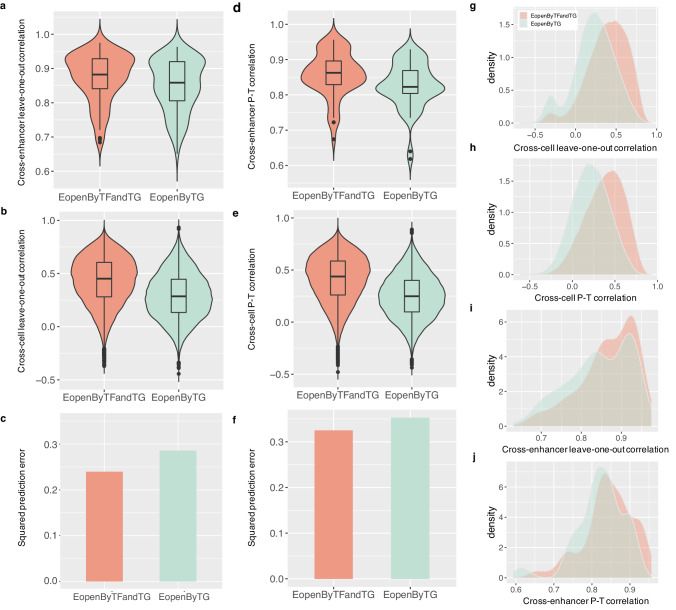
Comparison of different learning models on benchmark data. **a**–**c** The cross-enhancer and cross-cell correlation coefficients, and prediction errors from leave-one-out validation on 110 ENCODE cell lines based on different learning models. **d**–**f** The cross-enhancer and cross-cell P-T correlations, and prediction errors based on different learning models on 57 ENCODE test cell lines. **g**–**j** The distribution of cross-cell, and cross-enhancer PCCs based on level-one-out validation on 110 training cell lines, and 57 test cell lines. EopenByTG: using enhancer’s downstream genes’ expression only to predict its chromatin accessibility; EopenByTFandTG: using enhancer’s upstream binding TFs and downstream associated genes’ expression to predict its chromatin accessibility.

The PERD model (1) was designed to predict the enhancer’s chromatin activity by the transcriptional expression of its downstream associated genes and upstream binding TFs. To evaluate the performance of regression algorithms on learning, the cross-enhancer PCC, cross-cell PCC, and Squared prediction error were introduced. The leave-one-out cross-validation on 110 cell lines and validation on 57 test cell lines indicates that, comparing with elastic net and SVM, RF could achieve higher cross-cell, cross-enhancer PCCs, and smaller prediction errors (Supplementary Fig. 1). Since RF outperformed other two regression algorithms, we applied RF regression algorithm to implement regression learning in model (1), which called EopenByTFandTG, and compared it with only using downstream target gene’s expression to learn the enhancer’s openness (EopenByTG). As a result, EopenByTFandTG obtained higher cross-cell, cross-enhancer PCCs, and smaller prediction errors comparing with EopenByTG based on leave-one-out validation on 110 cell lines (Fig. 2a–c) and on 57 test cell lines (Fig. 2d–f). The distribution of cross-cell and cross-enhancer PCCs on leave-one-out validation on 110 cell lines (Fig. 2g, i) and on 57 test cell lines (Fig. 2h, j) also suggested better performance of EopenByTFandTG. That is, EopenByTFandTG outperformed EopenByTG consistently. In conclusion, the RF algorithm outperforms other two regression algorithms and enhancer’s chromatin activity prediction could be improved by including both upstream TFs’ and downstream genes’ transcriptional expression. Thus, in the rest of analysis, PERD model stands for using RF as regression model and using both upstream TF and downstream gene’s transcriptional expression to learn the enhancer’s chromatin accessibility.

The previous studies suggested the tissue/cell type specificity of enhancers^35–37^. We then validated the prediction results on a particular tissue/cell type based on leave-one-out validation on entire 167 ENCODE cell lines. The tissue types in validated dataset were shown in Supplementary Fig. 2, and the prediction results on tissue types with cell lines larger than 10 were shown in Supplementary Fig. 3. The cross-cell, cross-enhancer PCCs, and Squared prediction error exhibited significant variation across different tissue types. Muscle cells, the largest tissue type, achieved the highest cross-enhancer PCCs but lowest cross-cell PCCs, resulting in the worst prediction errors. These results suggested that, the prediction results were relied on the tissue types, but not determined by the number of cell lines in this tissue types.

### Characterization of the well predicted enhancers

**Fig. 3:**
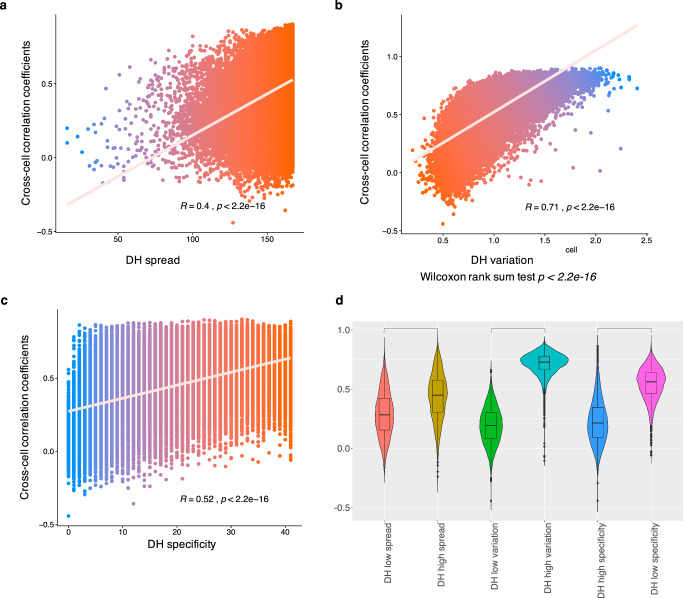
Characterizing enhancers that their accessibility can be well predicted. **a–c** The scatter plots showing the relationship between prediction results and enhancer properties (DH signal spread, variation, and specificity). **d** The boxplot shows the significant differences in prediction results for enhancers with different characteristics.

Both leave-one-out cross-validation on 110 training cell lines and predictions on 57 testing cell lines indicated relative lower across-cell P-T correlation than across-enhancer P-T correlation, suggesting that the prediction model performs well on a fraction of enhancers. For instance, based on leave-one-out cross-validation on whole 110 ENCODE cell lines, only about 50% (26892/54076) enhancers have their across-cell P-T correlation larger than 0.5. To summarize the characteristics for enhancers that would have better predictions, three properties derived from DNase-seq data (DH signal) were introduced, including the DH spread (defined by the number of cell lines with DH signal larger than 0), DH variation (defined by the standard deviation of DH signal across cell lines), and DH specificity (defined by the number of cell types with DH signal larger than 2). Based on predictions on 57 testing cell lines, we can see that, all three DH properties were correlated with the across-cell P-T correlation (Fig. 3a–c). The predictions significantly varied on different enhancer groups (Fig. 3d), meaning that the P-T correlation correlates with three DH properties. In particular, the enhancers that exhibit a broader distribution and greater variability in DH signal, are more likely to yield accurate prediction of chromatin accessibility.

### Evaluation of prediction model in an independent dataset

We then applied the PERD to an independent data (paired DNase-seq and RNA-seq data in Roadmap project) to further evaluate its prediction performance. In particular, the PERD was trained by the benchmark data, including paired DNase-seq and RNA-seq data in 167 ENCODE paired cell lines, and the enhancer’s openness was predicted based on transcriptional expression from Roadmap RNA-seq data. By comparing the predictions with the true openness value measured by Roadmap DNase-seq data, PERD’s prediction performance on new enhancers was evaluated. We found that PERD achieved over 0.5 across-enhancer P-T correlation and less than 0.4 prediction error (Supplementary Fig. 4A–C), indicating the great generalization of PERD in another independent scenario. The prediction results on tissue types in Roadmap data (Supplementary Fig. 2B) also suggested the tissue-specific prediction ability of PERD. Meanwhile, the correlation analysis between across-cell P-T correlation and DH characteristics indicates that the enhancers with high spread and variation along with low specificity might have better chance to get good predictions (Supplementary Fig. 4D).

### Revealing the drug responsive enhancers through predicting their chromatin accessibility changes

The evaluation of prediction model on both benchmark data and independent data suggested that enhancers with high spread and variation along with low specificity might have better chance to achieve good predictions. Thus, the enhancers with more than 100 active cell lines (DH > 0), variations larger than lower quantile, and more than 10 active cell types were remaining for further analysis. That is, there were a total of 9,340 enhancers remaining. The PERD was trained on the benchmark data, and the chromatin accessibility before/after drug treatment for 9340 enhancers were predicted based on CMAP transcriptional expression data and compared to identify drug responsive enhancers. In addition, considering the tissue-specificity of PERD, only the largest two cancer types (breast and prostate) (Supplementary Fig. 2C) were considered here. Particularly, using transcriptional expression data on breast and prostate cancer cell lines (MCF7 and PC3) before/after drug treatment to learn the enhancer’s openness before/after drug treatment, respectively. In general, comparing with benchmark data, there were less bio-samples in CMAP with active enhancers (DH > 0). However, the percentage of CMAP instances with active enhancers were about larger than 80% for all drugs (Supplementary Fig. 5A). It means that most of CMAP instances achieved predicted value for representing enhancer chromatin activity. In addition, the variations of predicted enhancer activities were comparable with that in benchmark data, just that the predicted value were relative lower than true DH value in training data (Supplementary Fig. 5B). These results indicated that the distribution of predicted value was roughly close to the true DH signal. We then detected the drug responsive enhancers by finding the significantly altered enhancers after drug treatment (diffEnhancers).

The count of differential enhancers (diffEnhancers) exhibited significant variations across different drugs in both breast and prostate cancer. For example, in breast cancer, the number of diffEnhancers ranged from 2 for chlorpromazine to 494 for LY-294002 (Supplementary Fig. 6A). In contrast, for prostate cancer, drugs genistein and wortmannin lacked sufficient instances for differential openness analysis, and fluphenazine did not present enhancers with a logFC exceeding 0.5 and a $p$ value below 0.001. Consequently, the count of diffEnhancers ranged from zero for the aforementioned three drugs to 235 for trichostatin A (Supplementary Fig. 6C). Furthermore, the quantities of diffEnhancers differed between two cancer types. For instance, genistein showed 86 diffEnhancers in breast cancer but none in prostate cancer. With the exception of LY-294002 and trichostatin A, the remaining drugs did not share diffEnhancers (Supplementary Fig. 7). These findings suggest that the presence of diffEnhancers is contingent upon the specific drug and disease context.

### PERD associates genetic variants with drug responsive enhancers

From PERD, the drug-dependent enhancers were revealed. That is, the existing pharmacogenomics resources, such as CMAP and CDS-DB, can be expanded to form a drug, gene, and enhancer drug mechanism network. Base on this network, various applications can be made. For instance, associating PERD predictions with the existing pharmacogenetic variants, to extend annotations of genetic variants from disease to drug level. To this end, several validations were implemented. Firstly, the predicted diffEnhancers were associated with TF motifs, and for both two cancer type, all 13 drugs’ diffEnhancers were linked with at least one TF motif, and some even had two thousand TF motifs, such as drug trichostatin related enhancer: chr17:48538779–48607552 in prostate cancer (Supplementary Figs. 6B, D), implying the potential regulatory role of these diffEnhancers.

**Fig. 4:**
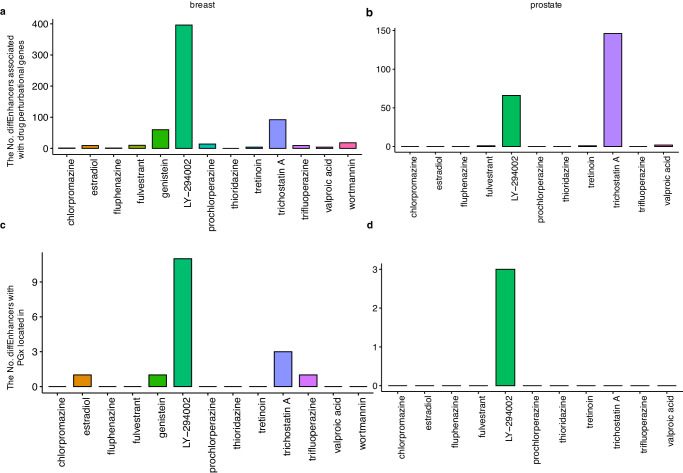
The application of PERD on CMAP data. The number of diffEnhancers that were associated perturbational genes for 13 investigated drugs in breast (**a**) and prostate cancer (**b**). The number of diffEnhancers with PGx located in for breast (**c**) and prostate cancer (**d**).

Then, the diffEnhancers were related to the drug perturbational genes, that is, find the overlap gene sets among diffEnhancers’ TGs and drug perturbational genes. Drug perturbational genes were defined as genes with significantly different expression level after drug treatment with *p* value less than 0.05 and absolute log transformed fold change larger than 0.8. As a results, for breast cancer, except for ‘thioridazine’, all other 12 drugs have at least one diffEnhancer associated with drug perturbational genes, and PI3K inhibitor ‘LY-294002’ even had 396 diffEnhancers with their TGs happening to be drug perturbational genes, which took about 80 percent of the total diffEnhancers (494) for ‘LY-294002’ (Fig. 4a). While, for prostate cancer, 5 out of 11 drugs have at least one diffEnhancer associated with drug perturbational genes, and potent Histone Deacetylase (HDAC) inhibitor ‘Trichostatin A’ had about 62% (146/235) diffEnhancers with their TGs also displaying significantly different expression level after ‘Trichostatin A’ treatment (Fig. 4c). All these results suggested that, PERD might uncover pharmacogenetic variants that will result in the perturbation of the corresponding gene expression. To further validate this assumption, the predicted diffEnhancers were linked to GTEx portal, which deposited over 20 thousand significant variant-gene associations based on permutations. Specifically, significant variant-gene associations were obtained from GTEx_Analysis_v8_eQTL.tar whole blood genes with q value less than 0.05, and diffEnhancers with variants associated with drug perturbed genes located in were reported. For breast cancer, 5 out of 13 drugs have at least one diffEnhancer associated with drug PGx, and ‘LY-294002’ had the most diffEnhancers with PGx located in (Fig. 4b). While, for prostate cancer, only ‘LY-294002’ got diffEnhancer with eQTLs for drug perturbed genes located in (Fig. 4d).

**Fig. 5:**
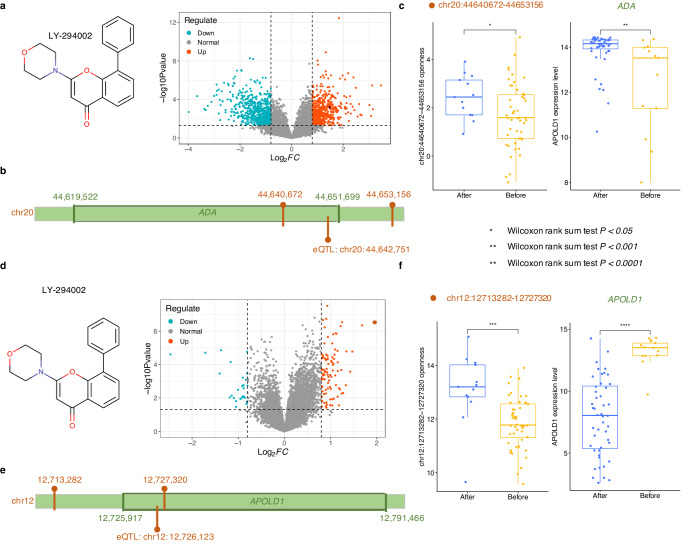
Two examples showing how PERD enhances the causality of drug perturbation by the candidate regulatory element of those drug associated genes. **a** LY-29400 and its diffEnhancers in breast cancer. **b** The genome positions of LY-29400’s diffEnhancer and its associated gene, and eQTL associated with LY-29400’s perturbed gene in breast cancer. **c** Comparison of the diffEnhancer’s chromatin accessibility and its associated gene’s expression level after LY-29400 treatment in breast cancer. **d** LY-29400 and its diffEnhancers in prostate cancer. **e** The genome positions of LY-29400’s diffEnhancer and its associated gene, and eQTL associated with LY-29400’s perturbed gene in prostate cancer. **f** Comparison of the diffEnhancer’s chromatin accessibility and its associated gene’s expression level after LY-29400 treatment in prostate cancer.

The detail of these diffEnhancers and variants for drug perturbed genes were investigated for potential candidates that are worthy for further experimental validation for two cancer types. Specifically, we checked if these variants associated genes were consistent with diffEnhancers’ TGs. Those variants that were associated with drug consistent perturbations were then associated with the diffEnhancers and listed in Supplementary Table 1. For instance, LY-294002 was linked with breast cancer by previous study^38^. Enhancer ‘chr20: 44, 640, 672–44, 653, 156’ was output as the diffEnhancer for LY-294002 (Fig. 5a). The variation in eQTL ‘chr20: 44, 642, 751’ would lead to the changes in the expression of gene adenosine deaminase (*ADA*), and located in the genome region of enhancer ‘chr20: 44, 640, 672–44, 653, 156’ (Fig. 5b). Enhancer ‘chr20: 44, 640, 672–44, 653, 156’ exhibited significantly different activity after LY-294002 treatment (Wilcoxon rank sum test *p* < 0.05, Fig. 5c). In addition, ‘chr20: 44, 640, 672–44, 653, 156’ associated gene *ADA* displayed the significantly different expression level after LY-294002 treatment (Wilcoxon rank sum test *p* < 0.001, Fig. 5c). All these results indicate that the variant in enhancer ‘chr20: 44, 640, 672–44, 653, 156’ would intervene LY-294002 sensitivity in breast cancer patients through altering the regulation of LY-294002 responsive gene: *ADA*.

Another example is LY-294002 in prostate cancer. LY-294002 was linked to prostate cancer by previous study^39^. Enhancer ‘chr12:12713282–12727320’ was output as the diffEnhancer for LY-294002 (Fig. 5d), and eQTL ‘chr12: 12,726,123’ was located in the genome region of ‘chr12:12713282–12727320’ (Fig. 5e). Enhancer ‘chr12:12713282–12727320’ displayed significantly different activity after LY-294002 treatment (Wilcoxon rank sum test *p* < 0.0001, Fig. 5d). In addition, both enhancer ‘chr12:12713282–12727320’ and eQTL ‘chr12: 12,726,123’ associated gene apolipoprotein L domain containing 1 (*APOLD1*) displayed the significantly different expression level after LY-294002 treatment (Wilcoxon rank sum test *p* < 0.0001, Fig. 5f). All these results provide supported evidences for the relationship between variants in ‘chr12:12713282–12727320’ and LY-294002’s sensitivity in prostate cancer patients. That is, the variant in enhancer ‘chr12:12713282–12727320’ would intervene LY-294002 sensitivity in prostate cancer patients through altering the regulation of LY-294002 perturbed gene: *APOLD1*.

To further validate the usefulness of PERD in revealing genetic variations that could intervene patient’ response to drug treatment, the CDS-DB database deposited the perturbations in patient-derived cancer cell was introduced. The application on CDS-DB breast cancer data revealed the diffEnhancers associated drug perturbed genes (Supplementary Table 2). Th further PGx was identified from PharmGKB database^40^. As a result, Celecoxib associated PGx was ‘rs4133101’ (chr5: 40, 679, 465), which will cause the variation in the expression of gene Prostaglandin E Receptor 4 (*PTGER4*) according to PharmGKB database. In addition, the expression level of *PTGER4* displayed the significant difference after Celecoxib treatment. PERD outputs enhancer ‘chr5: 40, 674, 520–40, 690, 311’ as the diffEhancer of Celecoxib, and the target gene of enhancer ‘chr5: 40, 674, 520–40, 690, 311’ was *PTGER4* (Supplementary Fig. 8A). The Docetaxel’s diffEnhancer ‘chr11:3, 958, 402–3, 961, 399’ was associated with perturbed gene Ribonucleotide Reductase Catalytic Subunit M1 (*RRM1*), and the variant ‘rs9937’ (chr11:4, 138, 227) related with clinical anticancer drug Docetaxel by PharmGKB database was located in gene body of *RRM1* (Supplementary Fig. 8B).

In summary, PERD offered a set of genetic variants related with regulatory region of drug perturbed genes, which would result in alterations in drug sensitivity through regulating those genes. This could provide rich information for personalized drug treatment.

## Discussion

In this work, we developed a computational framework, PERD, to identify regulatory elements associated with drug sensitivity. To this end, we firstly constructed a machine learning model to probe chromatin accessibility of enhancers based on transcriptional expression of their linked downstream genes and upstream binding TFs. The model was trained by the paired DNase-seq and RNA-seq data curated from ENCODE and Roadmap data resources. The results demonstrated the model’s efficacy in predicting enhancer openness with its potential enhancer-TF and enhancer-gene interactions. Subsequently, we applied the model to predict and compare enhancer openness pre- and post-administration of specific drugs. Enhancers exhibiting significant differences in openness upon treatment (referred to as diffEnhancers) were identified as drug responsive enhancers. The identified diffEnhancers were further related to TF motifs and PGx resources.

The variants linked with a given drug may provide a great opportunity for drug repurposing. For instance, Fulvestrant, a selective estrogen receptor degrader, was used to treat hormone receptor (HR)-positive metastatic breast cancer in postmenopausal women with disease progression as well as HR-positive^41^. PERD reported two associated enhancers (chr1:26529190–26536400 and chr6:30506802–30512599) with GWAS SNPs (rs112750178 and rs140668832) located in their genome regions. Both two variants were associated trait of “Cervical Cancer” by GWAS, suggesting the potential usage of Fulvestrant in treatment of cervical cancer. In a prior study, Fulvestrant previously used as a treatment for cervical cancer in mice^42^. The experiments indicated that Fulvestrant could efficiently clear cancer and its precursor lesions in both mice cervix and vagina^42^. All these findings provide the great chance of Fulvestrant in cervical cancer in human, and its potential usage in other gynecological cancers has been tested by a clinical trial (No. NCT03926936, started on March 13, 2019, estimated end by Dec. 31, 2025). Besides linking to GWAS, our predictions can be also associated with the individual patient whole genome data in future, to reveal the drug-dependent variants that could be a great target to affect the efficacy of clinical drug.

The validations on both ENCODE and Roadmap data indicated that the prediction results varied on different tissue types. That is, PERD is tissue-type specific. Here, PERD was only applied on the largest two cancer type in CMAP, breast cancer cells (MCF-7) and prostate cancer cells (PC3). The drug responsive enhancers varied a lot for these two cancer types (Supplementary Fig. 8), and the number of diffEnhancers depends on the number of instances for analysis. In future, we would apply PERD on a big pharmacogenomics resource, such as LINCS L1000^43^, to get more stable results.

DNase-I hypersensitive sites sequencing (DNase-seq) and Assays for Transposase-Accessible Chromatin sequencing (ATAC-seq) are two widely used protocols for genome-wide investigation of chromatin accessibility. DNase-seq and ATAC-seq are based on the use of cleavage enzymes (DNase-I, enzymes which hydrolyze phosphodiester bonds of DNA molecules, and Tn5, transposases, respectively), which recognize and cleave DNA in open chromatin regions. Comparing with DNase-seq, ATAC-seq requires fewer cells and is less laborious, and the number of ATAC-seq-based studies was higher than that of DNase-seq-based studies in recent years. PERD model can be used both in DNase-seq and ATAC-seq data with paired RNA-seq data. In future work, we will try to apply ATAC-seq to do the follow-up research. For instance, considering the fact that enhancers are highly cell-type or cell-state specific, we will conduct genome-wide investigation of chromatin accessibility before/after drug treatment to identify the causality of drug perturbation based on paired single-cell ATAC-seq and DNase-seq to address the issue of drug resistant caused by tumor heterogeneity.

## Methods

### The PERD model

#### Construction of enhancer-gene, and TF-enhancer network

The enhancer-gene network was extracted from GeneHancer, a database of genome-wide enhancer-to-gene and promoter-to-gene associations, embedded in GeneCards^44^. The enhancers were integrated from ENCODE, the Ensembl regulatory build, the functional annotation of the mammalian genome (FANTOM) project^45^, the VISTA Enhancer Browser^46^, etc. Using the enhancer-to-gene associations in GeneHancer, the enhancer-gene network was constructed.

The TF-enhancer network was constructed based on ENCODE human ChIP-seq data. In particular, the TF binding regions were firstly summarized from ENCODE human ChIP-seq data, and a TF with the binding site located in a given enhancer region was then associated with this enhancer. The enhancers’ genome regions were defined by GeneHancer database^46^.

### Learning enhancer’s chromatin accessibility from its associated genes and binding TFs’ expression

Once the enhancer-gene and enhancer-TF networks were constructed, the enhancer’s chromatin accessibility was then predicted by the following regularized regression model given paired expression and chromatin accessibility data across diverse cellular contexts:

1 $$\begin{array}{cc}\begin{array}{c}\min \\ \alpha ,\beta \end{array} & ||O-{\alpha }_{0}-{\beta }_{0}-{\gamma }_{T}\mathop{\sum}\limits_{p\in {E}_{T}}{\alpha }_{p}{{TF}}_{p}-{\gamma }_{G}\mathop{\sum}\limits_{q\in {E}_{G}}{\beta }_{q}{{TG}}_{q}||_{2}^{2}+\lambda \left({{||}\alpha {||}}_{2}^{2}+{{||}\alpha {||}}_{1}+{{||}\beta {||}}_{2}^{2}+{{||}\beta {||}}_{1}\right)\end{array}$$

where $O$ is chromatin accessibility value (openness) for a given enhancer (determined by the maximum DH signal along this enhancer region), ${{TF}}_{p}$ and ${{TG}}_{q}$ are the expression level for *p*th TF and *q*th gene associated with the given enhancer in the network, respectively, ${\gamma }_{T}$ and ${\gamma }_{G}$ are pre-defined parameters to represent the weight for TF and gene in prediction, respectively, ${E}_{T}$ and ${E}_{G}$ are enhancer’s binding TF and associated gene set, respectively, and $\lambda$ is a pre-defined parameter to represent the weight for regularization.

### Model implementation and evaluation

To implement the model (1) in a more efficient way, we set ${\gamma }_{G}={\gamma }_{T}$, the model (1) then became Elastic net regression model^47^, which was implemented by R “glnmet” package. The model (1) can be treated as other regression model using different regularized terms, such as Random Forest (RF)^48^ and Support Vector Machine (SVM)^49^, which can be implemented by R ‘randomForest’ and R ‘e107’ package, respectively. To further simplify the implementation procedure, the enhancers with more than two associated genes and binding TFs were remained for further analysis. Therefore, there are a total of 54,076 enhancers remaining for further investigation.

Suppose we have a total of *M* enhancers with at least two associated genes and binding TFs across *N* cells, let ${O}_{.m}=({O}_{1m},\ldots ,{O}_{{Nm}})$ represents the measured chromatin accessibility value (openness) for *m-*th enhancer across *N* cell lines, ${O}_{n.}=({O}_{n1},\ldots ,{O}_{{nM}})$ represents the measured openness in the n-th cell lines for M enhancers, and $\hat{O}$ represents the predicted openness value. The following three statistics were introduced to evaluate the performance of the prediction model:

1. cross-cell correlation: ${\tau }_{C}={cor}\left({O}_{.m},{\hat{O}}_{.m}\right)$;
2. cross-enhancer correlation: ${\tau }_{E}={cor}\left({O}_{n.},{\hat{O}}_{n.}\right)$;
3. squared prediction error: $\tau =\frac{\sum _{n}\sum _{m}{({O}_{{nm}}-{\hat{O}}_{{nm}})}^{2}}{\sum _{n}\sum _{m}{({O}_{{nm}}-\bar{O})}^{2}}$, where $\bar{O}$ is the mean openness for all *M* enhancers across *N* cells.

### Reporting summary

Further information on research design is available in the Nature Research Reporting Summary linked to this article.

### Supplementary information

Supplemental material

Reporting summary

## Data Availability

No new experimental data was generated as part of this study. The benchmark datasets for evaluation of PERD are from ENCODE RNA-seq and DNase-seq data^32^, which were available at https://www.nature.com/articles/s41467–017–01188-x#additional-information. It includes RNA-seq data and DNase-seq data in 167 paired samples. The DH signal was normalized for each genome bin by ENCODE, and the openness value of an enhancer is determined by the highest DH signal observed within the genome bins corresponding to that enhancer. RNA-seq data and DNase-seq data in randomly selected 110 paired samples for leave-one-out cross-validation, and remaining 57 samples were used for testing. The independent test dataset was also extracted in^32^, including RNA-seq data and DNase-seq data in 70 paired samples from Roadmap Epigenomics Project^50^. The perturbation transcriptional expression profiles came from a well-known pharmacogenomics resource, CMAP^33^, which were available at https://www.broadinstitute.org/connectivity-map-cmap. CMAP contains 6100 gene expression profiles of 4 cell lines treated with 1309 distinct small molecules with diverse doses. R package ‘limma’ was introduced to identify the significantly altered enhancers with logarithmic fold change (logFC) larger than 0.5 and *p* value smaller than 0.001. To achieve differently altered enhancers with more significance, drugs with more than 10 treatment dosages and two significant different types of treatment conditions were remaining for further analysis (Supplementary Table 3). Besides CMAP, CDS-DB^34^ was also introduced with patient-derived gene expression profiles induced by clinical drug treatment, which were available at http://cdsdb.ncpsb.org.cn/.

## Code availability

PERD has been developed into an open access software for research usage at https://github.com/wangyc82/PERD.
