# Supplementary material for: Identification of drug responsive enhancers by predicting chromatin accessibility change from perturbed gene expression profiles
Source: NPJ Syst Biol Appl. 2024 May 30;10:62. doi: 10.1038/s41540-024-00388-8 (PMC11139989; doi:10.1038/s41540-024-00388-8)
Supplement: Supplementary file 1 — Supplemental material [file 41540_2024_388_MOESM1_ESM.pdf]

Supplementary Materials

Identifying drug responsive enhancers by predicting chromatin accessibility change from perturbation gene expression profiles

Yongcui Wang[1*], Yong Wang[2,3*]

[1] State Key Laboratory of Phytochemistry and Plant Resources in West China, Kunming Institute of Botany, Chinese Academy of Sciences, Kunming, Yunnan, 650201, China

[2] CEMS, NCMIS, HCMS, MDIS, Academy of Mathematics and Systems Science, Chinese Academy of Sciences, Beijing, 100190, China

[3] Key Laboratory of Systems Biology, Hangzhou Institute for Advanced Study, University of Chinese Academy of Sciences, Chinese Academy of Sciences, Hangzhou, 330106, China

*Corresponding authors; E-mails: ywang@amss.ac.cn; wangyongcui@mail.kib.ac.cn.

Content:

Supplementary table 1

Predicted candidate enhancers, their associated drug perturbational genes, and PGx in CMAP data.

Supplementary table 2

Predicted candidate enhancers, their associated drug perturbational genes, and PGx in CDS-DB data.

Supplementary table 3

CMap drugs used for prediction.

Supplementary Figure 1

The prediction results (cross-enhancer, cross-cell PCCs, and squared prediction error) on 110 ENCODE training cells (A-C), and 57 test cells (D-F) based on different regression algorithms by either using target genes' expressions or both target genes and binding TFs' expressions.

Supplementary Figure 2

Tissue types on ENCODE, Roadmap, and CMap data (A-C). The tissue types shared by three data sets (D).

Supplementary Figure 3

The prediction results on large four tissue types. A, cross-enhancer prediction on leave-one-out-validation on all 167 ENCODE cells, B, cross-cell prediction on leave-out-validation on all 167 ENCODE cells, C, squared prediction error on leave-out-validation on all 167 ENCODE cells.

Supplementary Figure 4

The prediction results on Roadmap independent data. A, cross-enhancer prediction on entire data, and three largest tissue types, B, cross-cell prediction on entire data, and three largest tissue types, C, squared prediction error on entire data,

and three largest tissue types, D, The cross-cell PCCs on on different enhancer groups.

Supplementary Figure 5

A. The percentage of bio-samples with active enhancers (DH signal larger than 0);
B. The variations of enhancer openness after drug treatment.

Supplementary Figure 6

A,C. The number of diffEnhancers for breast and prostate cancer;
B,D. The number of diffEnhancers associated with TF motifs for breast and prostate cancer.

Supplementary Figure 7

The diffEnhancers shared by different cancer types for each single drug.

Supplementary Figure 8

A, The predicted diffEnhancer for clinical drug Celecoxib, and its associated perturbational gene and PGx;
B. The predicted diffEnhancer for clinical drug Docetaxel, and its associated perturbational gene and PGx.

Supplementary table 1

Drug	Candidate enhancers	eQTLs	Overlap genes among eQTLs associated drug perturbational genes and diffEnhancers' TGs	Cancer type
Estradiol	chr19:3760720-3763765	chr19 3761654	APBA3	breast
Genistein	chr19:17214422-17216612	chr19 17214972	USE1	breast
LY-294002	chr14:24441322-24443809	chr14 24443549	SDR39U1	breast
	chr19:18368031-18370247	chr19 18369799	GDF15	breast
	chr19:4398170-4404975	chr19 4402662	CHAF1A	breast
	chr17:64500923-64508621	chr17 64506317	DDX5	breast
	chr19:10224447-	chr19	MRPL4	breast

	10232352	10231337		
	chr17:76380995-76387032	chr17 76381613	SPHK1	breast
	chr17:48001202-48006399	chr17 48004549	NFE2L1	breast
	chr20:44518965-44523782	chr20 44521668	SERINC3	breast
	chr20:44640672-44653156	chr20 44642751	ADA	breast
	chr12:12713282-12727320	chr12 12726123	APOLD1	prostate
	chr1:42333201-42337730	chr1 42335364	FOXJ3	prostate
	chr6:43051727-43055143	chr6 43053781	CUL7	prostate
Trichostatin A	chr9:129773189-129778799	chr9 129773321	PTGES	breast
	chr17:78120560-78135339	chr17 78127382	TMC6	breast
Trifluoperazine	chr6:31493364-31498537	chr6 31494871	MICB	breast

Supplementary table 2

**Drug name**	**CDS-DB dataset signature ID**	**Sampl e szie**	**Drug type**	**No. of diffEnhance rs**	**No .of diffEnhance rs with TGs associated with drug sensitivity**	**No .of diffEnhance rs associated with PGx**
Atorvastati n	CDS_dataset_Microarray_ 107	48	Targeted therapy	12	1	0
Everolimus	CDS_dataset_Microarray_ 115	26	Targeted therapy	4	0	0
	CDS_dataset_Microarray_ 114	16	Targeted therapy			
Trastuzum ab	CDS_dataset_Microarray_ 53	84	Immunothera py	4	0	0
	CDS_dataset_Microarray_ 52	4	Immunothera py			

	CDS_dataset_Microarray_54	12	Immunotherapy			
Celecoxib	CDS_dataset_Microarray_63	44	Targeted therapy	30	6	1
Ruxolitinib	CDS_dataset_RNAseq_20	4	Targeted therapy	--		
Telapristone acetate	CDS_dataset_RNAseq_27	62	Targeted therapy	197	74	0
Docetaxel	CDS_dataset_Microarray_94	14	Chemotherapy	31	4	1

Supplementary table 3

**Name**	**Annotation**	**No. of instances before treatment**	**No. of instances after treatment**
Estradiol	A form of estrogen	22	15
Fulvestrant	Used to treat some hormone-related breast cancer	12	28
Genistein	A phytoestrogen	2	15
Chlorpromazine	Treats psychotic disorders such as schizophrenia	13	6
Fluphenazine	Treats schizophrenia and different types of behavior problems	4	14
LY-294002	PI3K inhibitor	13	48
Trichostatin A	A potent and reversible inhibitor of Histone Deacetylase (HDAC)	140	42
Thioridazine	Treats the symptoms of schizophrenia	6	14
Valproic acid	Treats seizures	36	21
Wortmannin	Commonly used as PI3K inhibitor	16	2
Tretinoin	Treats acne and other skin conditions	18	4

Prochlorperazine	Treat severe nausea and vomiting	4	12
Trifluoperazine	Treat anxiety or schizophrenia	4	12

Supplementary Figure 1

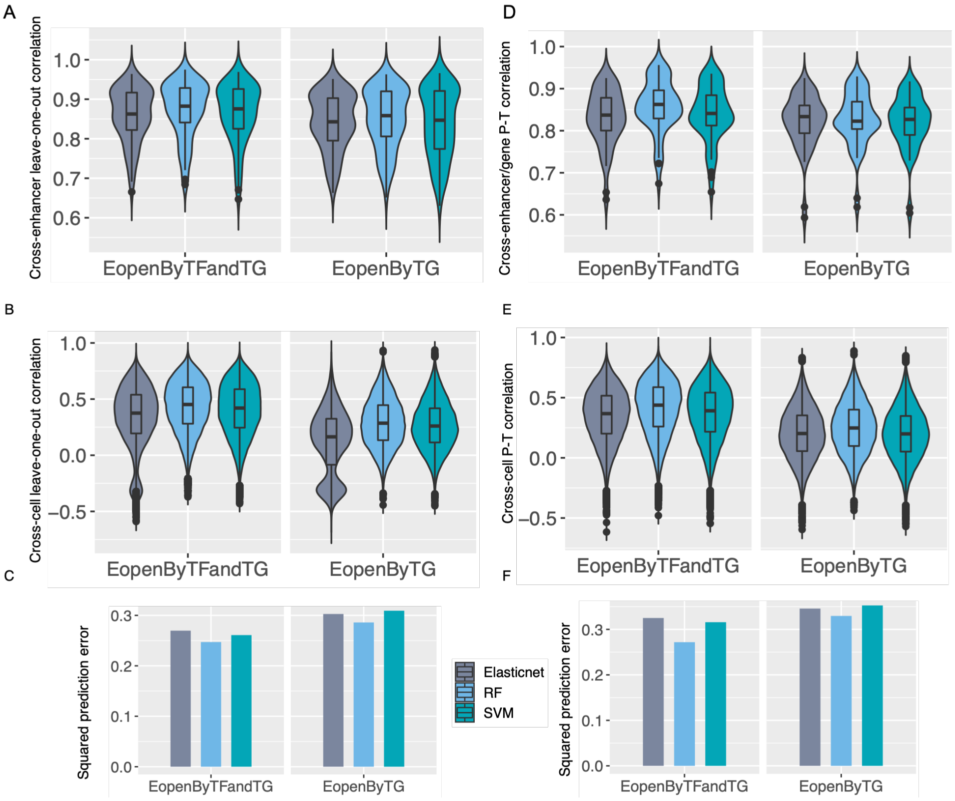

Supplementary Figure 2

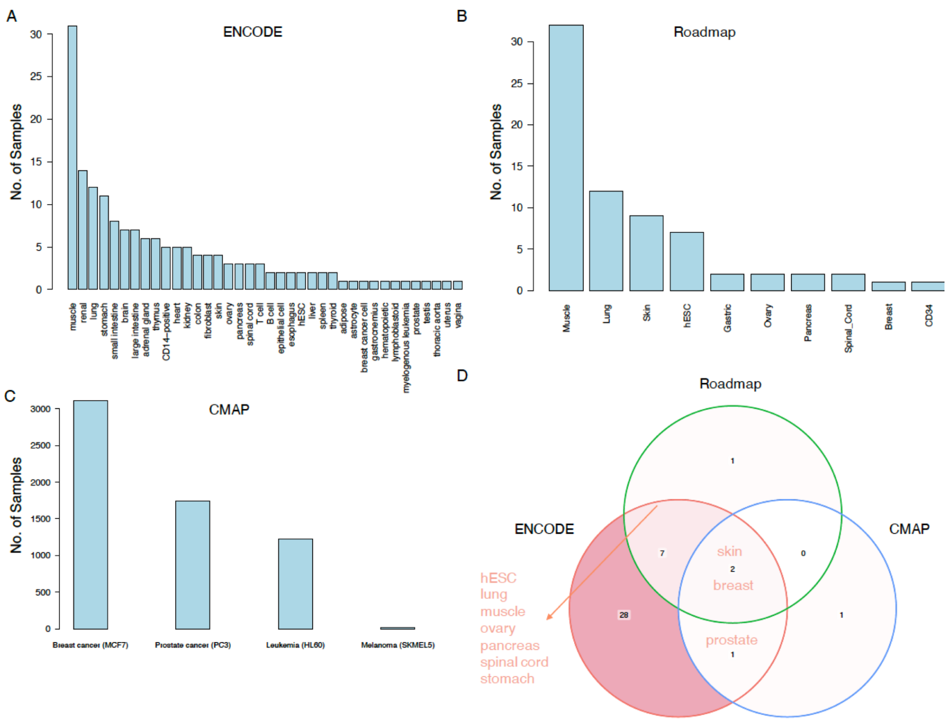

Supplementary Figure 3

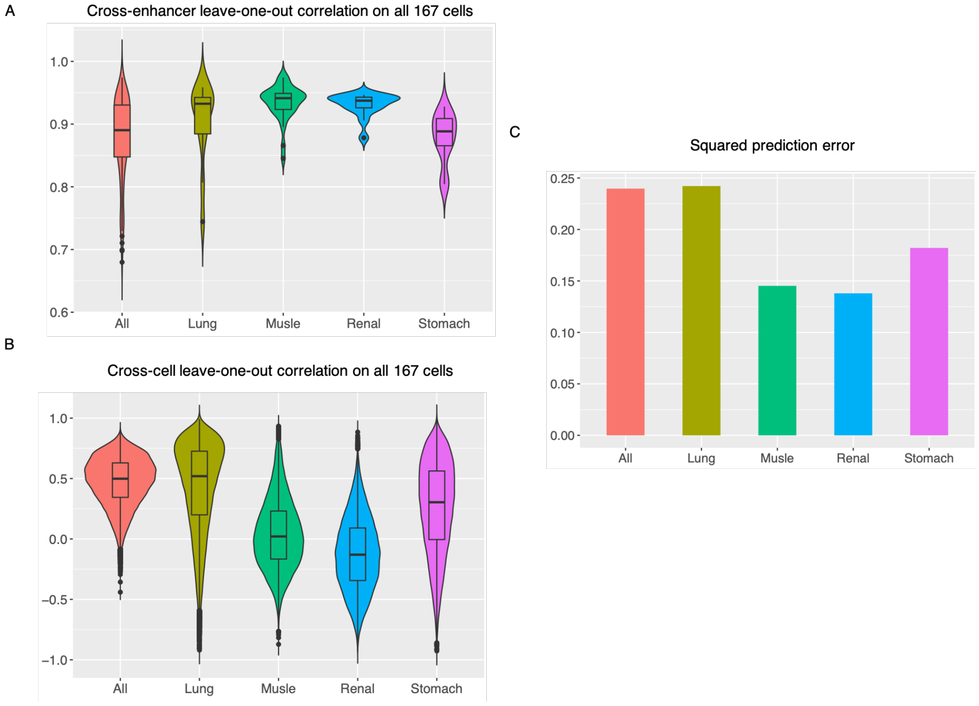

Supplementary Figure 4

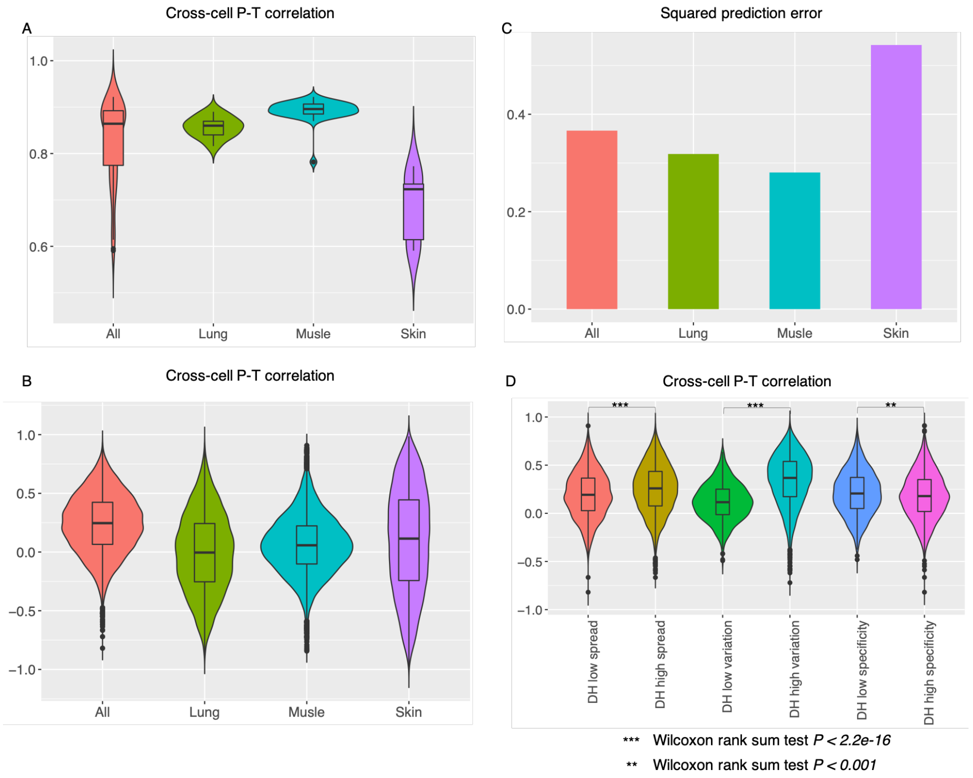

Supplementary Figure 5

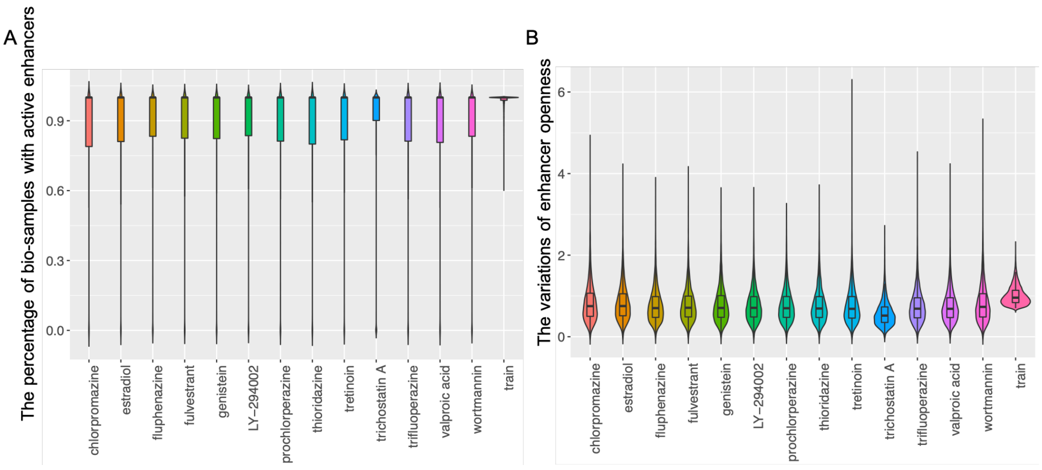

Supplementary Figure 6

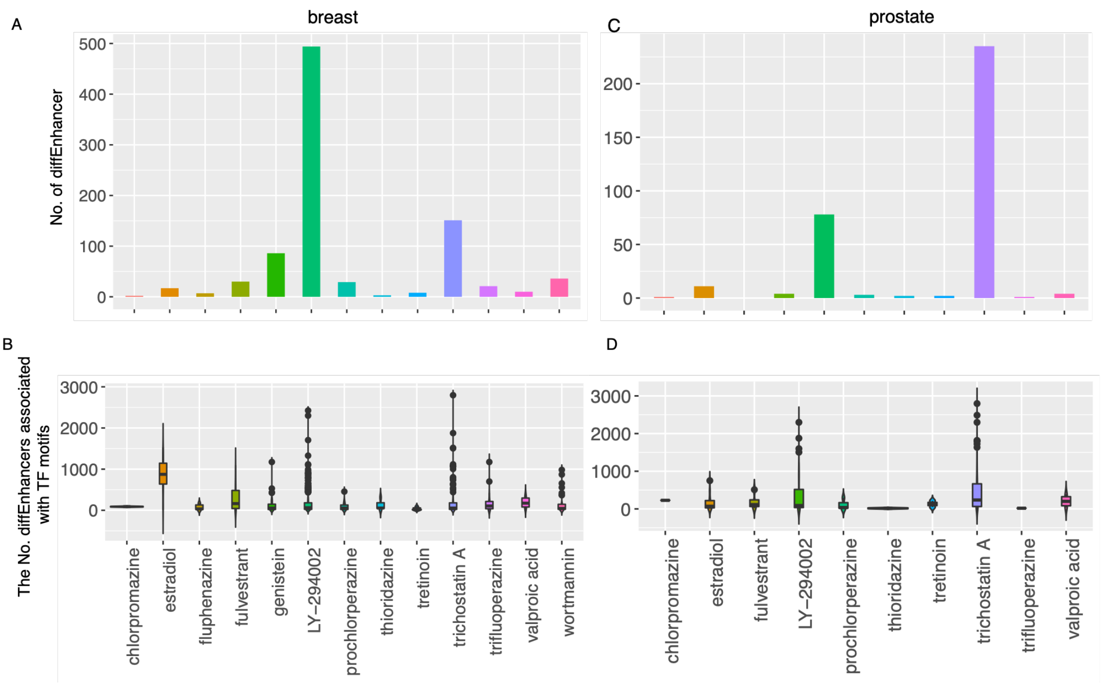

Supplementary Figure 7

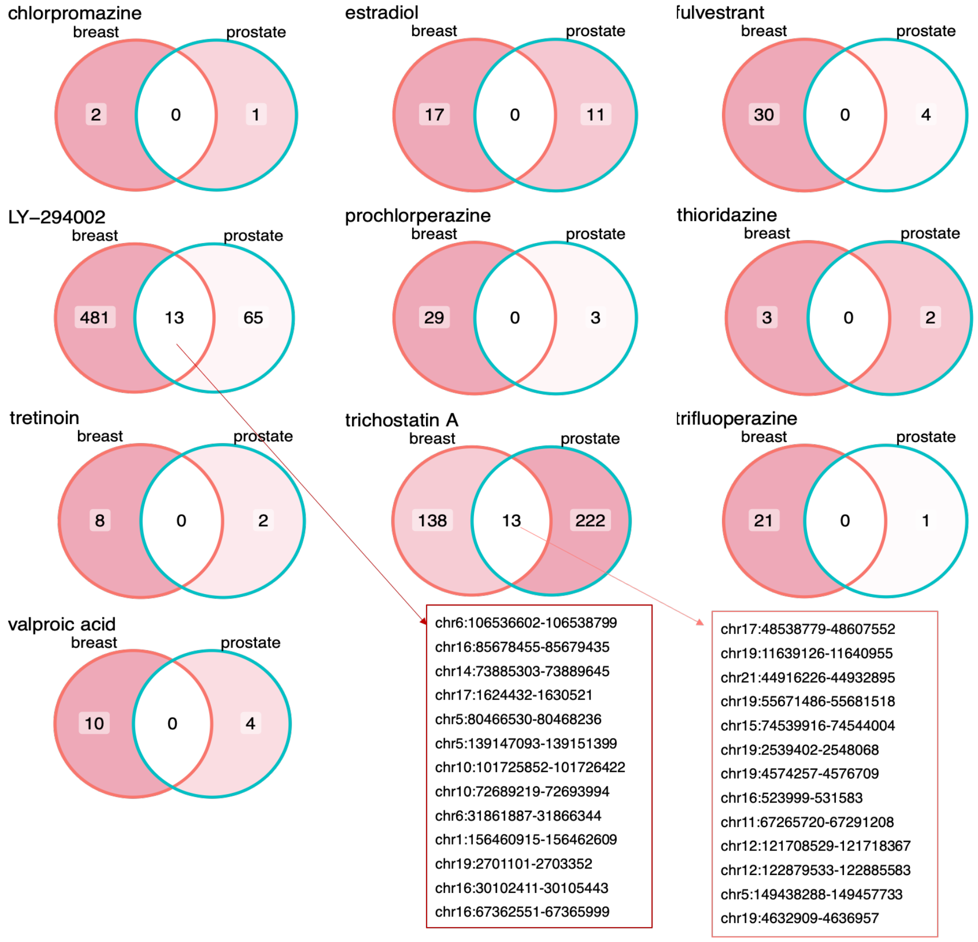

Supplementary Figure 8

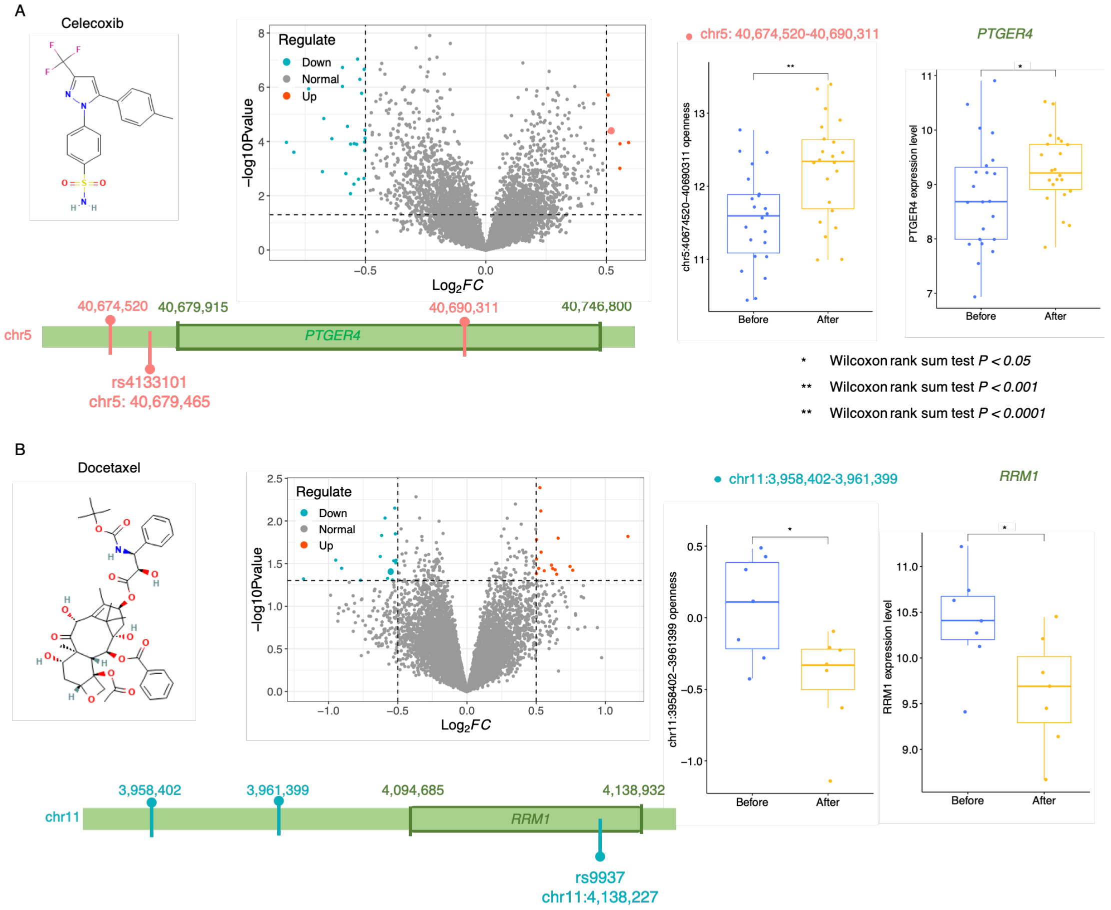

A
Celecoxib
Regulate
Down
Normal
Up
−log10Pvalue
Log2FC
chr5: 40,674,520-40,690,311
PTGER4
Before
After
40,674,520
40,679,915
40,690,311
40,746,800
chr5
rs4133101
chr5: 40,679,465
* Wilcoxon rank sum test P < 0.05
** Wilcoxon rank sum test P < 0.001
** Wilcoxon rank sum test P < 0.0001
B
Docetaxel
chr11:3,958,402-3,961,399
RRM1
3,958,402
3,961,399
4,094,685
4,138,932
chr11
rs9937
chr11:4,138,227
