# Supplementary material for: Identification of drug responsive enhancers by predicting chromatin accessibility change from perturbed gene expression profiles
Source: NPJ Syst Biol Appl. 2024 May 30;10:62. doi: 10.1038/s41540-024-00388-8 (PMC11139989; doi:10.1038/s41540-024-00388-8)
Supplement: Supplementary file 2 — Reporting summary [file 41540_2024_388_MOESM2_ESM.pdf]

Corresponding author(s): Yong Wang and Yongcui Wang
NPJSBA-01320R

Last updated by author(s): Apr 22, 2024

Reporting Summary

Nature Portfolio wishes to improve the reproducibility of the work that we publish. This form provides structure for consistency and transparency in reporting. For further information on Nature Portfolio policies, see our Editorial Policies and the Editorial Policy Checklist.

Statistics

For all statistical analyses, confirm that the following items are present in the figure legend, table legend, main text, or Methods section.

n/a	Confirmed	
☐	☒	The exact sample size (*n*) for each experimental group/condition, given as a discrete number and unit of measurement
☒	☐	A statement on whether measurements were taken from distinct samples or whether the same sample was measured repeatedly
☐	☒	The statistical test(s) used AND whether they are one- or two-sided<br>*Only common tests should be described solely by name; describe more complex techniques in the Methods section.*
☐	☒	A description of all covariates tested
☐	☒	A description of any assumptions or corrections, such as tests of normality and adjustment for multiple comparisons
☐	☒	A full description of the statistical parameters including central tendency (e.g. means) or other basic estimates (e.g. regression coefficient) AND variation (e.g. standard deviation) or associated estimates of uncertainty (e.g. confidence intervals)
☐	☒	For null hypothesis testing, the test statistic (e.g. *F*, *t*, *r*) with confidence intervals, effect sizes, degrees of freedom and *P* value noted<br>*Give P values as exact values whenever suitable.*
☒	☐	For Bayesian analysis, information on the choice of priors and Markov chain Monte Carlo settings
☒	☐	For hierarchical and complex designs, identification of the appropriate level for tests and full reporting of outcomes
☐	☒	Estimates of effect sizes (e.g. Cohen's *d*, Pearson's *r*), indicating how they were calculated

Our web collection on statistics for biologists contains articles on many of the points above.

Software and code

Policy information about availability of computer code

Data collection	The benchmark datasets for evaluation of PERD are from ENCODE paired RNA-seq and DNase-seq data. It includes RNA-seq data and DNase-seq data in 167 paired samples. An independent test dataset included RNA-seq data and DNase-seq data in 70 paired samples from Roadmap Epigenomics Project. The perturbation transcriptional expression profiles from CMAP that contains 6,100 gene expression profiles of 4 cell lines treated with 1,309 distinct small molecules with diverse doses.
Data analysis	The DH signal was normalized for each genome bin by ENCODE, and the openness value of an enhancer is determined by the highest DH signal observed within the genome bins corresponding to that enhancer. We randomly selected RNA-seq data and DNase-seq data in 110 paired samples for leave-one-out cross-validation, and remaining 57 samples for testing. R package `limma' was introduced to identify the significantly altered enhancers with logarithmic fold change (logFC) larger than 0.5 and p-value smaller than 0.001. To achieve differently altered enhancers with more significance, drugs with more than 10 treatment dosages and two significant different types of treatment conditions were remaining for further analysis. PERD model has been developed into an open access software for research usage at https://github.com/wangyc82/PERD.

For manuscripts utilizing custom algorithms or software that are central to the research but not yet described in published literature, software must be made available to editors and reviewers. We strongly encourage code deposition in a community repository (e.g. GitHub). See the Nature Portfolio guidelines for submitting code & software for further information.

Data

Policy information about availability of data

All manuscripts must include a data availability statement. This statement should provide the following information, where applicable:

- Accession codes, unique identifiers, or web links for publicly available datasets
- A description of any restrictions on data availability
- For clinical datasets or third party data, please ensure that the statement adheres to our policy

No new experimental data was generated as part of this study. RNA-seq and DNase-seq data used here were from ENCODE and RoadMap and are available at https://www.nature.com/articles/s41467-017-01188-x#additional-information. The pharmacogenomic data were from CMAP and are available at https://www.broadinstitute.org/connectivity-map-cmap. Patient-derived gene expression profiles were from CDS-DB and are available at http://cdsdb.ncpsb.org.cn/.

Research involving human participants, their data, or biological material

Policy information about studies with human participants or human data. See also policy information about sex, gender (identity/presentation), and sexual orientation and race, ethnicity and racism.

Reporting on sex and gender	This study did not include any human participants or human data.
Reporting on race, ethnicity, or other socially relevant groupings	This study did not include any human participants or human data.
Population characteristics	This study did not include any human participants or human data.
Recruitment	This study did not include any human participants or human data.
Ethics oversight	This study did not include any human participants or human data.

Note that full information on the approval of the study protocol must also be provided in the manuscript.

Field-specific reporting

Please select the one below that is the best fit for your research. If you are not sure, read the appropriate sections before making your selection.

☒ Life sciences ☐ Behavioural & social sciences ☐ Ecological, evolutionary & environmental sciences

For a reference copy of the document with all sections, see nature.com/documents/nr-reporting-summary-flat.pdf

Life sciences study design

All studies must disclose on these points even when the disclosure is negative.

Sample size	All data in this study were from public resources. The benchmark datasets for evaluation of PERD are from ENCODE paired RNA-seq and DNase-seq data. It includes RNA-seq data and DNase-seq data in 167 paired samples. An independent test dataset included RNA-seq data and DNase-seq data in 70 paired samples from Roadmap Epigenomics Project. The perturbation transcriptional expression profiles from CMAP that contains 6,100 gene expression profiles of 4 cell lines treated with 1,309 distinct small molecules with diverse doses.
Data exclusions	No data was excluded.
Replication	No replication data
Randomization	All data was preprocessed before publication. No more randomization was conducted.
Blinding	Data collection was performed by Dr Yongcui Wang, and no more other guys participated this part of work.

Reporting for specific materials, systems and methods

We require information from authors about some types of materials, experimental systems and methods used in many studies. Here, indicate whether each material, system or method listed is relevant to your study. If you are not sure if a list item applies to your research, read the appropriate section before selecting a response.

Materials & experimental systems

n/a	Involved in the study
☒	☐ Antibodies
☒	☐ Eukaryotic cell lines
☒	☐ Palaeontology and archaeology
☒	☐ Animals and other organisms
☒	☐ Clinical data
☒	☐ Dual use research of concern
☒	☐ Plants

Methods

n/a	Involved in the study
☒	☐ ChIP-seq
☒	☐ Flow cytometry
☒	☐ MRI-based neuroimaging

Plants

Seed stocks	No plants data here.
Novel plant genotypes	No plants data here.
Authentication	No plants data here.
